# Pattern recognition of Hodgkin-Huxley equations by auto-regressive Laguerre Volterra network

**DOI:** 10.1186/1471-2202-16-S1-P156

**Published:** 2015-12-18

**Authors:** Kunling Geng, Vasilis Z Marmarelis

**Affiliations:** 1 Biomedical Engineering Department, University of Southern California, Los Angeles, CA 90089, USA; 2 Biomedical Simulations Resource, Los Angeles, CA 90089, USA

## 

**Figure 1:**
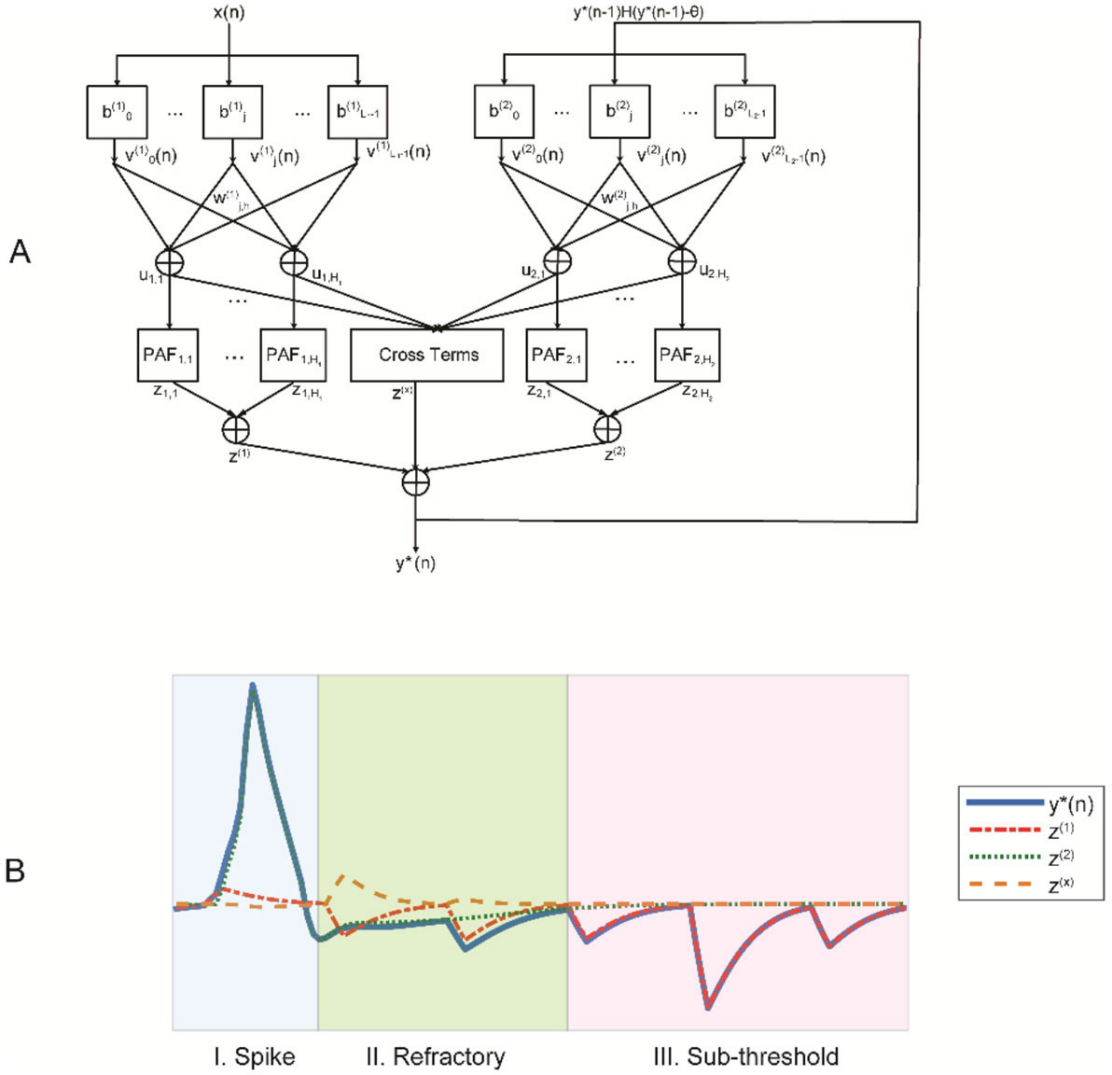
A Structure of ASLVN for modeling H-H equations, where the input x(n) is the randomly injected current and the output y*(n) is the membrane potential. **B **The predictions results, z^(1) ^represents the exogenous output, z^(2) ^represents the autoregressive output and z^(x) ^represents the cross term output.

A nonparametric, data-driven nonlinear auto-regressive Volterra (NARV) [1] model has been successfully applied for capturing the dynamics in the generation of action potentials, which is classically modeled by Hodgkin-Huxley (H-H) equations. However, the compactness still need to be improved for further interpretations. Therefore, we propose a novel Auto-regressive Sparse Laguerre Volterra Network (ASLVN) model (shown in Figure 1A), which is developed from traditional Laguerre Volterra Network (LVN) and principal dynamic mode (PDM) framework [2].

We adopt stochastic global optimization algorithm Simulated Annealing [3] to train the ASLVN instead of Back-propagation method [2] to avoid local minima and convergence problems. We also use lasso regularization [4] to enhance the spasity of the network and prune redundant branches for parsimony. The prediction results are shown in Fig.1B, it can be seen that the exogenous output z^(1) ^represents the subthreshold dynamics in phase III, and the autoregressive output z^(2) ^dominates in the spike shape in phase I, and the cross term output z^(x) ^helps to maintain the refractory period by cancelling the effect of z^(1) ^in phase II and we also observe that refractory inhibition effect decays after initiation of AP, which explains the absolute refractory period and relative refractory period in physiology.
